# Homozygous familial hypercholesterolemia with stenosis of the left anterior descending coronary artery successfully treated with weekly low‐density lipoprotein apheresis for 16 years without percutaneous coronary intervention

**DOI:** 10.1002/ccr3.2299

**Published:** 2019-07-16

**Authors:** Takanori Yasu, Masahiro Shimoyama, Hiroshi Wada, Tomohiro Iwakura, Shigeru Toyoda, Atsuhiko Kawabe, Takushi Sugiyama

**Affiliations:** ^1^ Department of Cardiovascular Medicine and Nephrology Dokkyo Medical University Nikko Medical Center Nikko Japan; ^2^ Yuai Clinic Saitama Japan; ^3^ Department of First Integrated Medicine 1, Saitama Medical Center Jichi Medical University Saitama Japan; ^4^ Department of Cardiovascular Surgery Sakakibara Heart Institute Tokyo Japan; ^5^ Department of Cardiovascular Medicine Dokkyo Medical University Mibu Japan

**Keywords:** angina, apheresis, homozygous familial hypercholesterolemia, iodine allergy

## Abstract

We successfully treated a patient with homozygous familial hypercholesterolemia (HoFH) with stable coronary arterial disease using optimal medical therapy and low‐density lipoprotein (LDL) apheresis for 16 years without percutaneous coronary intervention or bypass surgery. Intensive LDL lowering using apheresis and medication protected the patient from coronary atherosclerotic progression even in HoFH.

## 1 INTRODUCTION

Familial hypercholesterolemia (FH) is an autosomal disorder of lipid metabolism characterized by remarkably elevated low‐density lipoprotein cholesterol (LDL‐C) levels, cutaneous xanthomas, and a family history of premature atherosclerosis. Prevalence of homozygous (HoFH) and heterozygous FH is 1:160 000 and 1:250, respectively.^1^, ^2^, ^3^ The cholesterol exposure burden in HoFH greatly increases the risk of atherosclerotic cardiovascular disease and premature death. We successfully treated a patient with homozygous familial hypercholesterolemia (HoFH) with stable coronary arterial disease by intensive LDL‐C lowering using optimal medical therapy and weekly LDL apheresis for 16 years without percutaneous coronary intervention or bypass surgery.

## 2 CASE REPORT

A 47‐year‐old woman with HoFH was referred to our institute to continue biweekly LDL apheresis and appropriate lipid‐lowering medication in 1998. She had a history of multiple tendinous, tuberous, interdigital xanthomas since 1966, at the age of 10 years, and underwent repeated resection of the xanthomas. In 1971, she was diagnosed with hyperlipidemia. In 1990, at the age of 34 years, serum total cholesterol, LDL‐C, high‐density lipoprotein cholesterol, triglyceride, and lipoprotein (a) levels were 1438, 1401, 27, 51, and 96 mg/dL, respectively. Her mother and father had hypercholesteremia, and her mother died of acute myocardial infarction at the age of 83 years and father died of pneumonia at the age of 51 years. Two of her sisters also had hypercholesteremia. The clinical manifestations were typical of those associated with HoFH, and the family history was also compatible with the syndrome. In 1990, she was started on biweekly LDL apheresis for HoFH using dextran sulfate cellulose columns (Liposorber LA‐15; Kaneka Medical Products) to selectively remove apolipoprotein B‐containing lipoproteins from the plasma.^4^, ^5^ The mean pre‐/postweekly apheresis LDL‐C levels/2 months decreased from 201.4 ± 10.1 mg/dL (*P* < 0.0001 vs baseline) to 21.2 ± 5.1 mg/dL (*P* < 0.00001 vs pre‐apheresis).

**Figure 1:**
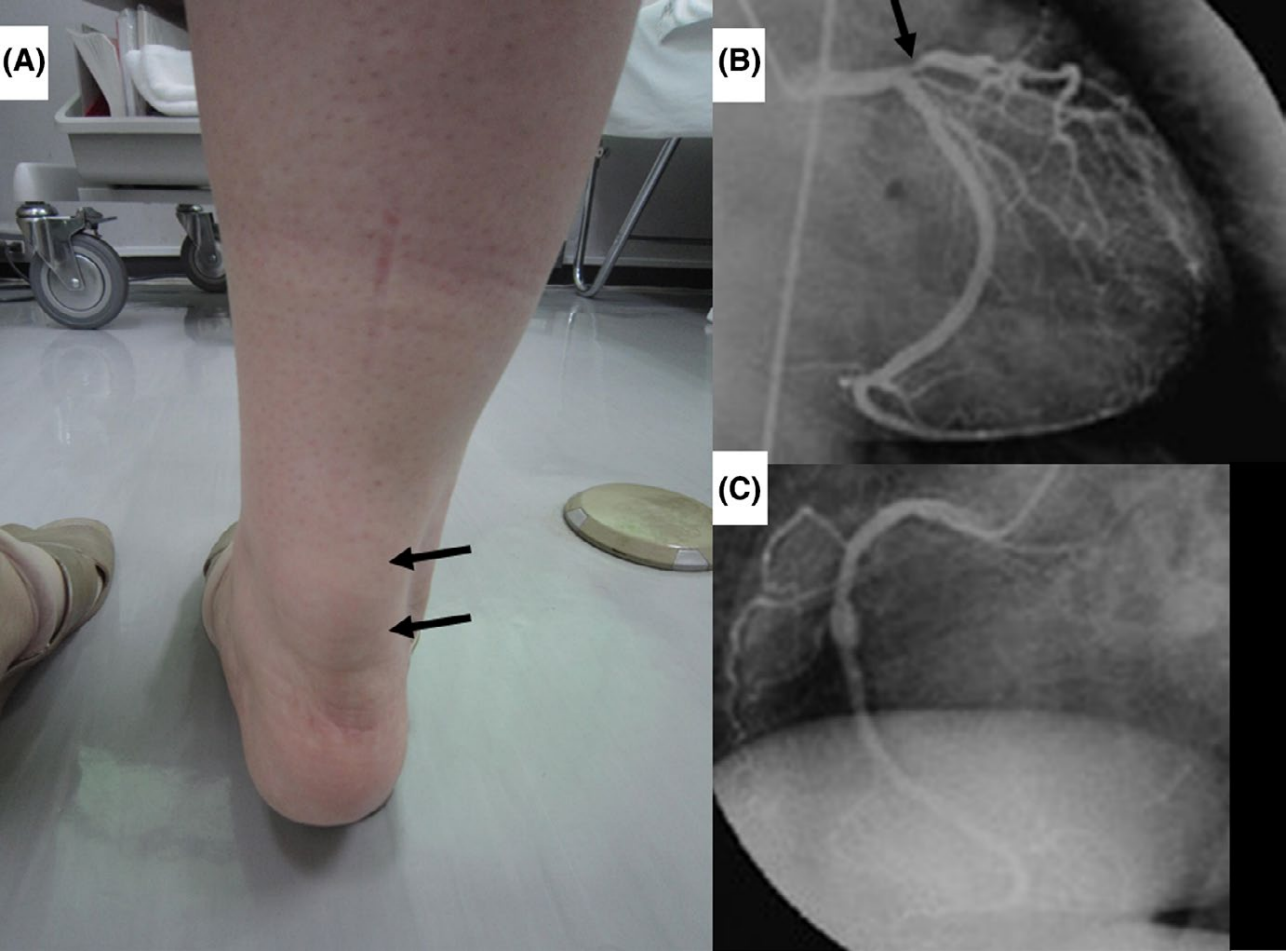
Remarkable swelling of the Achilles tendon, 31‐mm thickness (panel A, arrows) due to tendinous xanthoma. Coronary arteriography in 1999 revealed severe stenosis (arrow) in the proximal left anterior descending artery (panel B) and moderate stenosis in the midportion of the right coronary artery (panel C)

Her body mass index was 22.3 kg/m^2^, blood pressure was 110/67 mm Hg, heart rate was 70 bpm, and systolic ejection murmur was Levine grade III/VI in the second right sternal border referred to neck in March 1990. There was no audible crackle in the lungs. Her Achilles tendons were very swollen, to 31 mm (normal value <9mm), due to tendon xanthoma (Figure 1A); additional xanthomas were found in the upper eyelids, elbows, and knees. The Master two‐step exercise stress electrocardiogram revealed −1.0‐mm horizontal ST depression in the leads V_4‐6_ with chest tightness. She began experiencing chest tightness on exertion. In May 1999, coronary arteriography revealed severe stenosis (90%) in the proximal left anterior descending (LAD) artery (Figure 1B) and 50% stenosis in the midportion of the right coronary artery (Figure 1C). An ultrasound cardiogram revealed mild aortic stenosis with normal left ventricular contraction. She rejected coronary bypass surgery. Unfortunately, she had severe allergic reactions, including asthma and dermatitis, to iodine, aspirin, and cilostazol, and she also rejected percutaneous coronary intervention. Therefore, we increased the frequency of the LDL apheresis from biweekly to weekly in order to inhibit systemic atherosclerotic progression. Intensive medication therapy (atorvastatin 40 mg/d, colestimide 3 g/d, probucol 1000 mg/d, bezafibrate 400 mg/d, bisoprolol 5 mg/d, and nicorandil 15 mg/d) was continued. The maximum dose of atorvastatin is 40 mg/d in Japan. The mean pre‐/postweekly apheresis LDL‐C levels decreased from 170.3 ± 15.1 mg/dL (*P* < 0.01 vs prebiweekly apheresis) to 19.2 ± 5.0 mg/dL. Her angina symptoms significantly improved. She underwent the Master two‐step exercise stress electrocardiogram every year, and ST changes after the test remained unchanged compared with the first one conducted in 1999. She experienced no cardiovascular events and died at the age of 59 years due to pancreatic cancer in July 2015.

## 3 DISCUSSION

HoFH is a very rare, but exceedingly aggressive, form of FH. Most individuals with HoFH experience severe coronary heart disease and aortic stenosis by their mid‐20s.^2^, ^4^, ^5^ According to the most recent observational study, over 90% of individuals with FH are not appropriately diagnosed.^1^ Treatments for HoFH include a maximal dose of a strong statin such as atorvastatin, ezetimibe, bile acid‐binding resins, and LDL apheresis and/or proprotein convertase subtilisin‐kexin type 9 inhibitors.^3^, ^4^, ^5^, ^6^ In Japan, the maximum doses of atorvastatin, rosuvastatin, and pitavastatin are 40, 20, and 4 mg/d, respectively, even for cases of familial hypercholesterolemia, whereas in the United States and Europe, the maximum dose of atorvastatin is 80 mg/d. As treatment goals, the European Atherosclerosis Society, NCEP/ATPIII guidelines, International FH Foundation, and Japan Atherosclerosis Society suggest that LDL‐C levels <100 mg/dL in adults or <70 mg/dL in adults with clinical coronary artery disease or diabetes should be targeted.^6^, ^7^

Low‐density lipoprotein apheresis improves the ischemic limbs seen in patients with peripheral arterial occlusive disease via the upregulation of vascular endothelial growth factor (VEGF) and insulin‐like growth factor 1 associated with decreased fibrinogen levels^8^ and by reducing reactive oxygen species production in the leukocytes.^9^ Our data suggest that aggressive control of LDL‐C with apheresis and medications for patients with HoFH is an essential and a very promising method for preventing the progression of ischemic heart disease and aortic stenosis in cases where revascularization cannot be applied. In this case, the woman with HoFH had no cardiovascular events for 16 years because of (a) very aggressive LDL‐C control with weekly LDL apheresis and medications, (b) appropriate drug compliance, (c) walking 4 days a week, (d) no smoking, and (e) no diabetes mellitus or hypertension. In addition to decreasing the plasma LDL‐C levels, LDL apheresis lowers plasma fibrinogen, lipoprotein(a), and C‐reactive protein levels, all of which may be risk factors for atherosclerosis.^1^, ^2^, ^3^ In fact, in our patient, plasma fibrinogen, lipoprotein(a), and C‐reactive protein levels were decreased with a single session of LDL apheresis from 400.0, 20.0, and 0.9 mg/dL to 220.0, 6.1, and 0.11 mg/dL, respectively. However, a recent Mendelian randomization study denied that CRP levels are associated with the development of cardiovascular events.^10^ Bosch et al^11^ conducted a retrospective analysis of 18 patients who underwent chronic LDL apheresis and revealed that angina and dyspnea as well as patients’ general status and subjective well‐being improved significantly. The objective cardiovascular event rate decreased from a total of 26% in the 3‐year period before LDL apheresis to 6% during the mean follow‐up of 3.8 years during chronic LDL apheresis therapy. Thus, the average event rate of 0.48 per patient‐year at baseline could be significantly reduced to 0.09 (*P* < 0.004) with LDL apheresis.^11^ The Italian Multicenter Study on Low‐Density Lipoprotein Apheresis (IMSLDLa‐WG/2) involving 18 centers in 2009, treating 66 men and 35 women with hypercholesterolemia, reported that ischemic cardiovascular events were not observed in any patient over 9 ± 6 years of treatment.^12^

In conclusion, we presented the case of a patient with HoFH, angina pectoris, severe stenosis of the LAD, mild aortic stenosis, and episodes of severe allergic reactions to aspirin and iodine who was successfully treated with weekly LDL apheresis and intensive lipid‐lowering agents as well as lifestyle modifications, without any cardiovascular events for 16 years.

## CONFLICT OF INTEREST

None declared.

## AUTHOR CONTRIBUTIONS

TY, MS, HW, TI, and AK: treated the patients. TY and MS: analyzed the data and wrote the manuscript. TI, ST, and TS: edited the manuscript.
